# Effects of Spatial Expression of Activating Transcription Factor 4 on the Pathogenicity of Two Phenotypes of Bovine Viral Diarrhea Virus by Regulating the Endoplasmic Reticulum-Mediated Autophagy Process

**DOI:** 10.1128/spectrum.04225-22

**Published:** 2023-03-20

**Authors:** Jing Wang, Ke-Yuan Chen, Sheng-Hua Wang, Yi Liu, Yi-Qing Zhao, Lan Yang, Guang-Hui Yang, Xiao-Jia Wang, Yao-Hong Zhu, Jin-hua Yin, Jiu-Feng Wang

**Affiliations:** a College of Veterinary Medicine, China Agricultural University, Beijing, China; b OIE Porcine-Reproductive and Respiratory Syndrome Reference Laboratory, China Animal Disease Control Center, Beijing, China; c College of Animal Science and Technology, Tarim University, Alar, China; David Geffen School of Medicine at UCLA

**Keywords:** bovine viral diarrhea virus, ER stress response, UPR, autophagy, viral replication

## Abstract

The endoplasmic reticulum (ER) stress response is a highly conserved stress-defense mechanism and activates the adaptive unfolded protein response (UPR) to mitigate imbalance. The ER stress-activated signaling pathways can also trigger autophagy to facilitate cellular repair. Bovine viral diarrhea virus (BVDV) utilizes the host cellular ER as the primary site of the life cycle. However, the interplay between cellular ER stress and BVDV replication remains unclear. This report reveals that cytopathic (cp) and noncytopathic (ncp) BVDV have distinct strategies to regulate UPR mechanisms and ER stress-mediated autophagy for their own benefit. Immunoblot analysis revealed that cp and ncp BVDV differentially regulated the abundance of ER chaperone GRP78 for viral replication, while the protein kinase RNA-like ER kinase (PERK)-eukaryotic translation initiation factor 2 subunit α (eIF2α)-activating transcription factor 4 (ATF4) pathway of the UPR was switched on at different stages of infection. Pretreatment with ER stress inducer promoted virion replication, but RNA interference (RNAi) knockdown of ATF4 in BVDV-infected cells significantly attenuated BVDV infectivity titers. More importantly, the effector ATF4 activated by cp BVDV infection translocated into the nucleus to mediate autophagy, but ATF4 was retained in the cytoplasm during ncp BVDV infection. In addition, we found that cp BVDV core protein was localized in the ER to induce ER stress-mediated autophagy. Overall, the potential therapeutic target ATF4 may contribute to the global eradication campaign of BVDV.

**IMPORTANCE** The ER-tropic viruses hijack the host cellular ER as the replication platform of the life cycle, which can lead to strong ER stress. The UPR and related transcriptional cascades triggered by ER stress play a crucial role in viral replication and pathogenesis, but little is known about these underlying mechanisms. Here, we report that cytopathic and noncytopathic BVDV use different strategies to reprogram the cellular UPR and ER stress-mediated autophagy for their own advantage. The cytopathic BVDV unconventionally downregulated the expression level of GRP78, creating perfect conditions for self-replication via the UPR, and the noncytopathic BVDV retained ATF4 in the cytoplasm to provide an advantage for its persistent infection. Our findings provide new insights into exploring how BVDV and other ER-tropic viruses reprogram the UPR signaling pathway in the host cells for replication and reveal the attractive host target ATF4 for new antiviral agents.

## INTRODUCTION

Bovine viral diarrhea virus (BVDV) is an enveloped, single-stranded RNA virus that has resulted in worldwide economic losses due to persistent infection and reproductive disorders. BVDV can exhibit two distinct phenotypes (biotypes) in *in vitro* cell culture: cytopathogenic (cp) or noncytopathogenic (ncp), which is one of its special characteristics (1). Noncytopathogenic BVDV (ncp BVDV), the most prevalent type of clinical isolates of BVDV, is responsible for persistent infection (PI) of cattle that continuously excrete large amounts of the virus throughout their lives (2). The mucosal disease is caused when ncp BVDV is mutated to the homologous cytopathogenic BVDV (cp BVDV) in PI cattle (3). BVDV is an endoplasmic reticulum-tropic virus belonging to the *Flaviviridae* family, utilizing the endoplasmic reticulum (ER) as a replication platform for its life cycle. With the emergence of new subtypes and variants of the virus, further research is required to find some novel targets of vaccines and antiviral agents to prevent or combat BVDV. Therefore, better knowledge of the interplay of BVDV with host cells is crucial for determining its pathogenesis and developing therapeutic strategies.

The ER is an essential organelle that mediates several cellular functions, including protein synthesis, calcium homeostasis, and phospholipid synthesis (4, 5), and is also involved in a series of complex transcriptional cascades such as autophagy, apoptosis, inflammation, and innate immunity (6). Viral infection perturbs ER homeostasis and generates unavoidable ER stress, especially ER-tropic viruses such as the *Flaviviridae* family. During times of ER stress response, the unfolded protein response (UPR) contributes to the restoration of ER homeostasis (7). In mammalian cells, the peptide-folding environment is monitored by three protein sensors: activating transcriptional factor 6 (ATF6), inositol requiring enzyme 1 (IRE1), and PKR-like ER kinase (PERK) (7). Under ER stress conditions, these sensors were selectively activated by the glucose regulated protein 78 (GRP78; also known as HSPA5 or BiP). GRP78 acts as a constitutively expressed ER-resident chaperone and a UPR regulator. When GRP78 is upregulated, it means that the UPR program is initiated. The three UPR sensors regulated by GRP78 induce different subsequent downstream pathways to alleviate ER stress. Activated ATF6 exposes Golgi localization signals and migrates to the Golgi membrane. Subsequently, ATF6 is cleaved by two proteases (8), and its cytoplasmic domain regulates the expression of GRP78 and protein disulfide isomerases (9). The other two sensors, PERK and IRE1, undergo homodimerization and autophosphorylation following GRP78 disassociation (10). Phosphorylated PERK (p-PERK) continues to phosphorylate the eukaryotic translation initiation factor 2 subunit α (eIF2α, also referred to as EIF2S1). Phosphorylated eIF2α (p-eIF2α) effectively attenuates translation and can also induce the expression of activating transcription factor 4 (ATF4). As a typical transcriptional activator, ATF4 controls the transcription of multiple proteins that determine cell fate (10). Lastly, phosphorylated IRE1 has endonuclease activity and splices of multiple mRNAs destined for the ER, which relieves the global translation pressure of the ER. Although most target mRNAs are degraded, phosphorylated IRE1 (p-IRE1) unconventionally splices the 26-bp nucleotide from the unspliced X-box-binding protein-1 (XBP1u) mRNA to yield XBP1 (XBP1s) (11, 12). XBP1s controls the transcription of some target genes involved in the ER degradation-enhancing alpha-mannosidase-like protein (EDEM) and chaperones (13, 14). Members of the *Flaviviridae* family usually manipulate the UPR signaling pathway to promote viral translation and persistence through the activation of one or more of the three arms of the UPR (15–17). The ER is also involved in a series of complex transcriptional cascades such as autophagy, apoptosis, inflammation, and innate immunity (6).

Autophagy is a highly conserved cellular recycling process involving the degradation of macromolecules and the recycling of the breakdown products. Previous research has revealed that autophagy is a prosurvival mechanism in the cell under stress conditions (e.g., nutrient deprivation, pathogen infection, and unfolded protein aggregation) via the formation of the autophagosome and fusion with the lysosome (18). The ER stress is critical in the regulation of autophagy (19) because the persistent ER stress can mediate the activation of autophagy through Ca^2+^, IRE1α, PERK, and ATF6 signal pathways (20). Moreover, each of the three effectors is involved at a different stage of autophagy. Several studies have shown that the PERK-eIF2α-ATF4 pathway is essential for the presence of ER stress-mediated autophagy machinery components, such as the microtubule-associated protein 1 light chain 3 (LC3) and p62 (also known as SQSTM1), especially the effector ATF4 (21, 22). The transducer ATF4 and C/EBP-homologous protein (CHOP) target promoters to allow the transcription of several genes involved in the formation and function of the autophagosome in response to ER stress (21, 23). Recent studies have shown the relationship between UPR and autophagy during infection by viruses belonging to the *Flaviviridae* family. The UPR modulators inhibit hepatitis C virus (HCV)-induced LC3-phosphatidylethanolamine conjugation (24); West Nile virus activates the UPR but does not cause autophagy (25). However, the cross talk between the UPR and autophagy is still not completely clear.

Previous studies have shown that cp BVDV actives the PERK branch of the UPR signaling pathway (26, 27), but the effect of this event on viral replication and the consequences of chronic ER stress signaling during ncp BVDV infection are unknown. Considering the critical role of the ER in the replication of *Flaviviridae* members, we aimed to explore the interplay of UPR signaling and ER stress-mediated autophagy with different BVDV phenotypes and also investigated how BVDV modulated the UPR for its own replication. In this report, we found that cp and ncp BVDV can deploy the downstream factor of the UPR signaling pathway to maximize replication efficiency. Moreover, the UPR induced by BVDV infection was associated with the activation of autophagy. Our findings emphasized the importance of the PERK-eIF2α-ATF4 pathway in persistent viral infection and provided a potential therapeutic target against BVDV.

## RESULTS

### Two phenotypes of BVDV adopt distinct strategies to induce ER stress.

**FIG 1:**
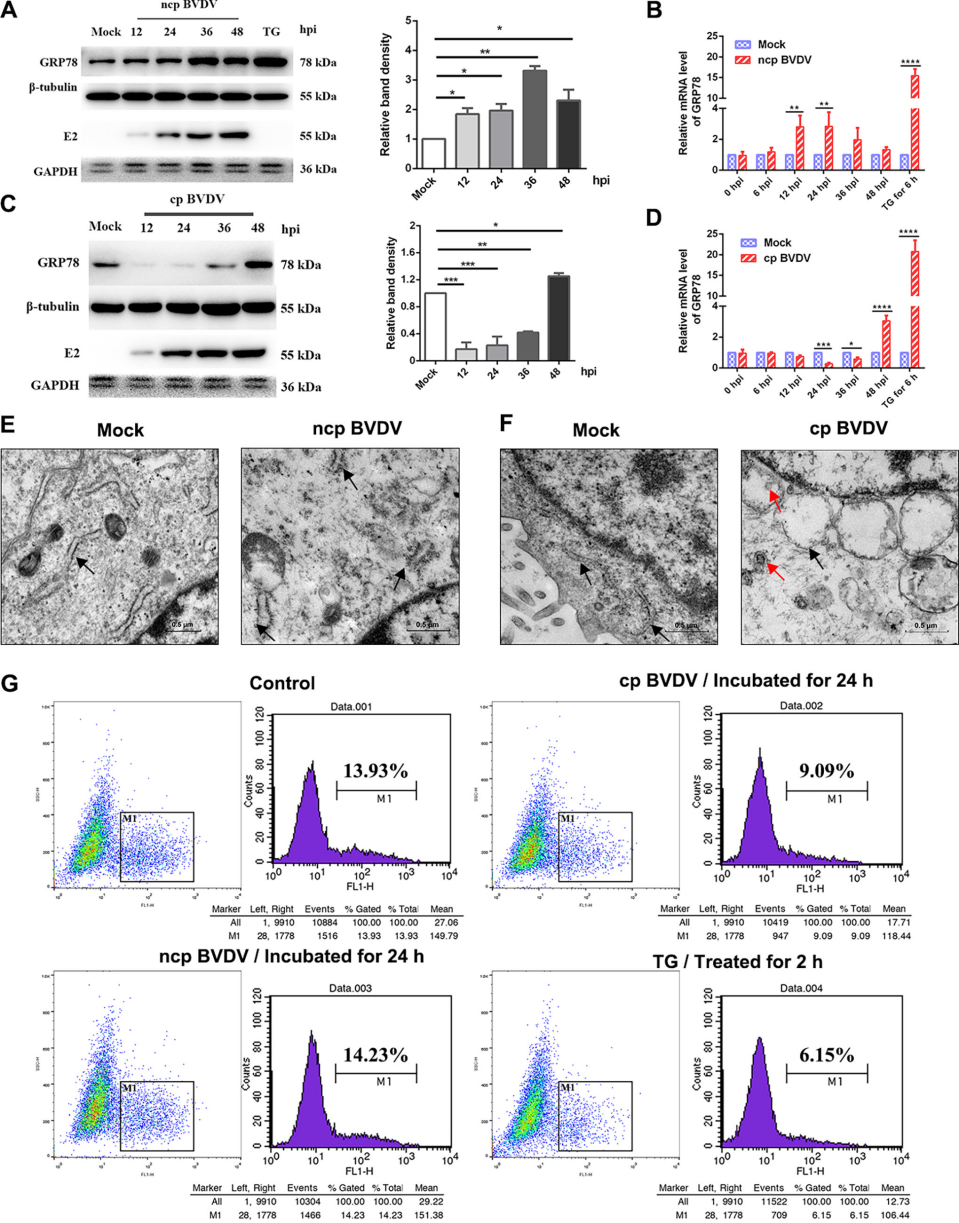
cp and ncp BVDV infections induce ER stress in MDBK cells. (A and C) The expression of GRP78 in MDBK cells infected with strain BVDV-BJ175170 (ncp BVDV) (A) and strain BVDV 1-NADL (cp BVDV) (C) at 12, 24, 36, and 48 hpi was analyzed by Western blotting. (Thapsigargin (TG) (200 nM)) treatment was identified as the positive control. β-Tubulin was set for the loading control. (B and D) The GRP78 mRNA levels were detected by RT-qPCR at the indicated time points after infection with strain BVDV-BJ175170 (ncp BVDV) (B) and strain BVDV 1-NADL (cp BVDV) (D). GRP78 mRNA levels in all groups were normalized to GAPDH prior to comparison between the infected and corresponding control groups. (E and F) The ER in MDBK cells was scanned by transmission electron microscopy at 36 h after infection with ncp (E) and cp BVDV (F). Black arrowheads, ER; red arrowheads, BVDV. Scale bar = 0.5 μm. (G) Flow cytometry analysis of Ca^2+^ flux in MDBK cells infected with each BVDV phenotype for 24 h. Cells incubated with TG (200 nM) for 2 h served as the positive control. MDBK cells incubated in DMEM for 24 h served as the negative control. One-way ANOVA or two-way ANOVA was adopted for statistical analysis. Values are presented as the mean ± SEM of three experiments that are independent and replicated, and the significance of data is indicated as follows: *, *P < *0.05; **, *P < *0.01; ***, *P < *0.001; ns, no significance.

To study the mechanism of cellular UPR induced by BVDV, we initially examined the expression abundance of the key marker (GRP78) of ER stress following BVDV 1-NADL standard strain (cp BVDV) and BVDV-BJ175170 isolate strain (ncp BVDV) infection in Madin-Darby bovine kidney (MDBK) cells. GRP78 is a resident ER chaperone, and its expression increases upon undergoing ER stress (28). For many enveloped and ER-tropic viruses, their proliferation produces large amounts of protein within the ER lumen; thus, host cells upregulate the expression of GRP78 to cope with the aggregated unfolded protein, which relieves the ER pressure (2, 29). We observed that GRP78 expression was gradually elevated in the case of ncp BVDV-infected cells (Fig. 1A). Interestingly, cp BVDV reduced GRP78 protein levels but subsequently increased the GRP78 protein level at 48 h postinfection (hpi), as shown by the determination of GRP78 protein (Fig. 1C). At the RNA level, two phenotypes showed trends similar to the respective protein expression levels (Fig. 1B and D). The ER stress inducer thapsigargin (TG) and tunicamycin (TM) were widely used as positive controls. TG induces ER stress by mobilizing calcium from the ER, while TM interferes with glycoprotein folding and maturation by blocking N-linked glycosylation in the ER. Treatment with TG resulted in a significant upregulation in GRP78 mRNA and protein expression levels (Fig. 1A to D). These data are further confirmed in Fig. S1A in the supplemental material.

As reported, a characteristic of the UPR response to ER stress is the induction of ER expansion. We examined cp BVDV-infected MDBK cells by transmission electron microscopy (TEM) and observed numerous dilated and vacuolated ERs, suggesting ER stress in MDBK cells (Fig. 1F, black arrows). Interestingly, in the case of ncp BVDV, the ER was not vacuolated but was slightly swollen and ruptured (Fig. 1E, black arrows). In contrast, the MDBK cells in the control group had a flat cystic ER.

The ER is a critical organelle that is a site for calcium storage (30). Several studies have documented that the virus may change the permeability of the ER to make calcium disturbances, which triggers ER stress (31). Thus, cells were loaded with Flou4-am dye in order to detect Ca^2+^ flow in the cytoplasm by flow cytometry. After cp BVDV incubation, a significant upregulation of Ca^2+^ levels was clearly observed (Fig. 1G), suggesting that cp BVDV disturbed Ca^2+^ homeostasis in the ER and thus may contribute to ER stress. Interestingly, incubation with ncp BVDV had no effect on cytoplasm Ca^2+^ levels. Additionally, TG was used as the positive control. We found that treatment with TG mobilized calcium from the ER (Fig. 1G), indicating that BVDV had the capacity to increase calcium levels. Together, the above-described results reveal that BVDV infection selectively activates Ca^2+^ signaling.

### cp and ncp BVDV activate the PERK pathway of UPR at different stages of infection.

**FIG 2:**
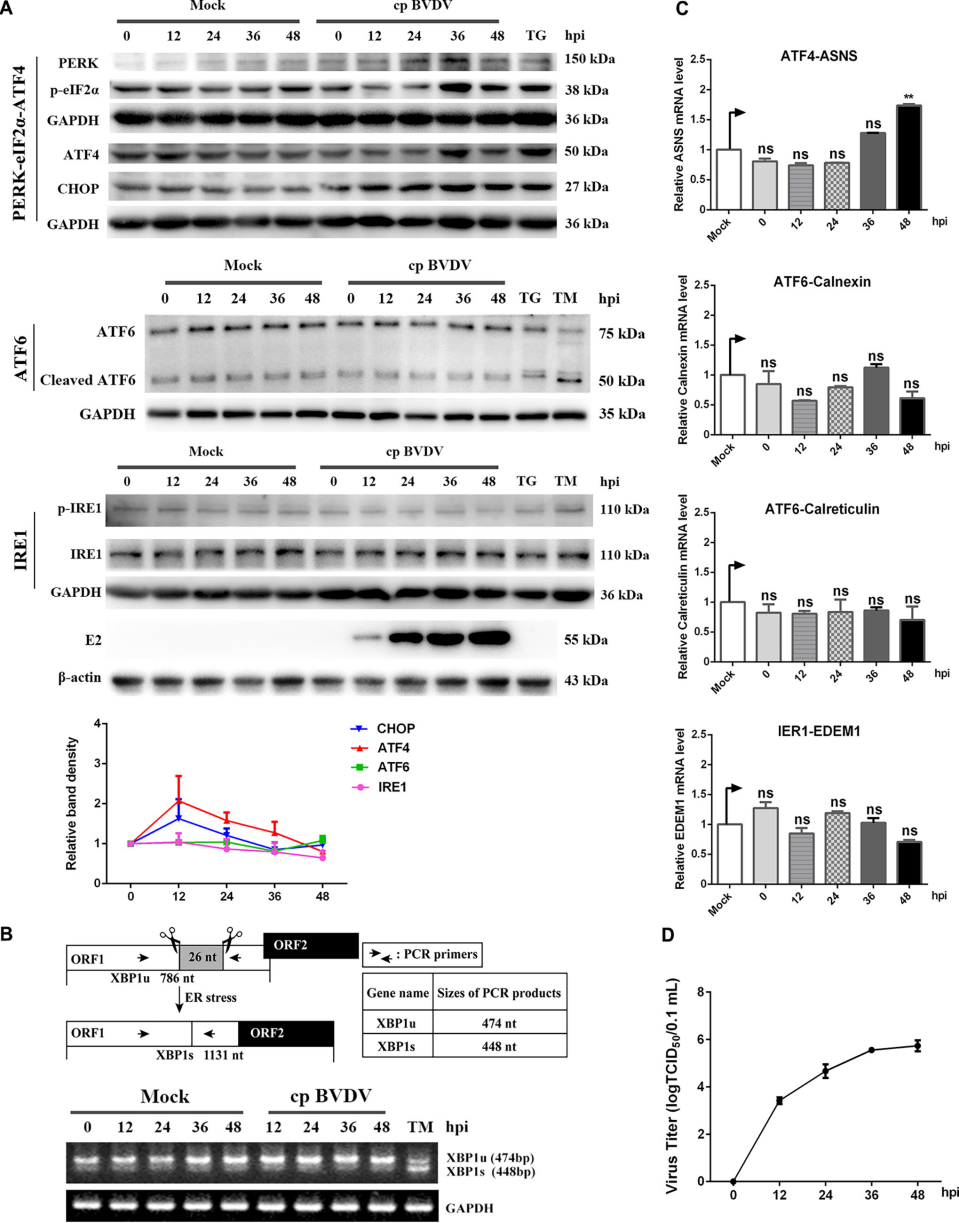
cp BVDV infection activates the PERK pathway at a later stage of infection. (A) Western blot analysis and relative intensity of UPR branching key proteins during cp BVDV infection. MDBK cells were treated with TM (5 μg/mL) and TG (200 nM) for 6 h or exposed to cp BVDV (MOI, 1) and harvested at 0, 12, 24, 36, and 48 hpi. Western blotting was conducted to analyze the cell lysates with the indicated antibodies. (B) Schematic diagram of XBP1 mRNA splicing and detection of XBP1 mRNA. The schematic diagram shows the splice site of XBP1 mRNA and the sizes of spliced and unspliced XBP1 after RT-PCR amplification. The amplification products of XBP1 of cp BVDV-infected cells were separated by electrophoresis on a 3% agarose gel. (C) Measurement of copy numbers of the PERK pathway downstream ASNS gene, the IRE1 pathway downstream EDEM1 gene, and the ATF6 pathway downstream calnexin and calreticulin genes by RT-qPCR in cp BVDV-infected cells. (D) Kinetics of cp BVDV production in MDBK cells at an MOI of 1. Student’s *t* test or one-way ANOVA was used for statistical analysis. Values are presented as the mean ± SEM of three experiments that are independent and replicated, and the significance of data is indicated as follows: *, *P < *0.05; ns, no significance.

Unlike the other viruses, cp BVDV infections caused a reduced level of GRP78 (Fig. 1C). Conceptually, it seemed likely that cp BVDV infection might activate the three branches of the UPR because GRP78 bonded to the three sensors and made them inactive. However, it was also possible that the three branches were not affected. In order to investigate this, the key UPR components were analyzed by Western blotting. The activation of the PERK-eIF2α-ATF4 branch of the UPR was observed in MDBK cells (Fig. 2A). In particular, the eIF2α was phosphorylated steadily, and the expression of PERK, ATF4, and CHOP kept increasing. In contrast, the expression levels of ATF6, cleaved-ATF6, and phosphorylation of IRE1 remained unchanged during infection, suggesting that neither the ATF6 nor the IRE1 branch was activated. Reverse transcriptase PCR (RT-PCR) was conducted to measure the XBP1 mRNA levels. Generally speaking, unspliced XBP1 (XBP1u) mRNA is specifically cleaved by IRE1 to produce a 26-nucleotide (nt) segment and then creates the spliced form of XBP1 (XBP1s) transcription factor (32). Results showed that both XBP1s and XBP1u remained unchanged at the mRNA level (Fig. 2B), indicating that IRE1 was not activated upon cp BVDV infection. In order to further analyze the IRE1-XBP1s and ATF6 signaling pathways, we measured the downstream regulatory genes of IRE1-XBP1s and ATF6 by quantitative reverse transcription-PCR (RT-qPCR), such as EDEM1 (a downstream regulatory gene of XBP1s) and calreticulin and calnexin (downstream genes of ATF6) during cp BVDV infection. The results showed that EDEM1 gene, calreticulin, and calnexin mRNA levels were all not modified in cp BVDV-infected MDBK cells, indicating that viral infection did not activate the ATF6 and XBP1 signaling pathways. However, the mRNA level of the asparagine synthetase (ASNS) gene (the canonical ATF4 target gene) was elevated (Fig. 2C), which was consistent with the upregulation of ATF4 protein. Collectively, our results demonstrate that cp BVDV infection activates the PERK-eIF2α-ATF4 signaling pathway.

**FIG 3:**
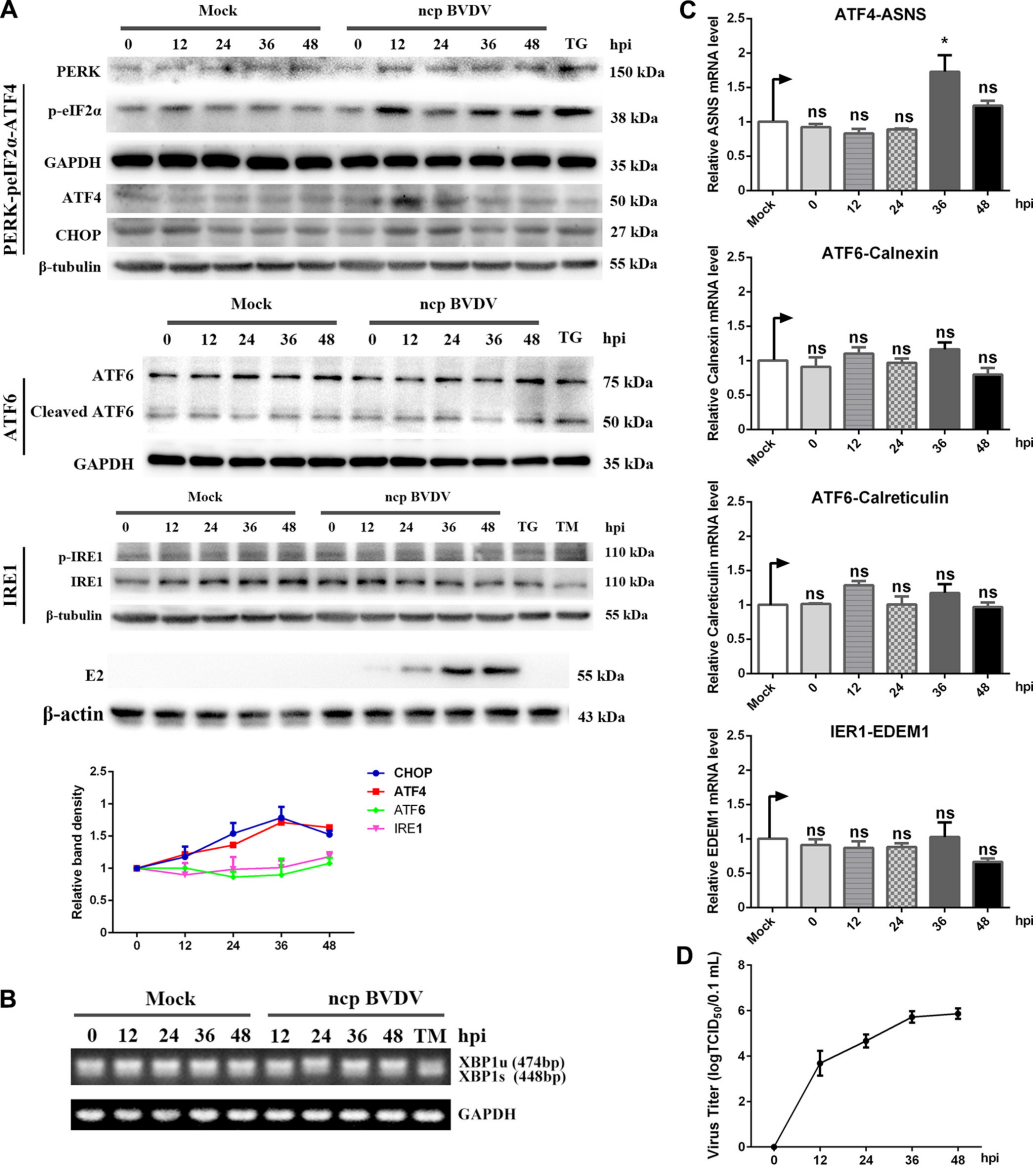
ncp BVDV activates the PERK pathway of UPR at an early stage of infection. (A) Western blot analysis and relative intensity of UPR branching key proteins during ncp BVDV infection. MDBK cells were treated with TM (5 μg/mL) and TG (200 nM) for 6 h or exposed to cp BVDV (MOI, 1) and harvested at 0, 12, 24, 36, and 48 hpi. Western blot analysis was conducted to analyze the cell lysates with the indicated antibodies. The results were obtained by probing the same PVDF membrane with the indicated antibodies or by stripping the membrane and reprobing it with the additional antibodies. Therefore, the blots labeled “β-tubulin” are identical. (B) Detection of spliced and unspliced XBP1 mRNA. The amplification products of XBP1 of cp BVDV-infected cells were separated by electrophoresis on a 3% agarose gel. (C) Measurement of copy numbers of the PERK pathway downstream ASNS gene, the IRE1 pathway downstream EDEM1 gene, and the ATF6 pathway downstream calnexin and calreticulin genes by RT-qPCR in ncp BVDV-infected cells. (D) Kinetics of ncp BVDV production in MDBK cells at an MOI of 1. Student’s *t* test or one- way ANOVA was used for statistical analysis. Values are presented as the mean ± SEM of three experiments that are independent and replicated, and the significance of data is indicated as follows: *, *P < *0.05; ns, no significance.

To better understand the molecular pathogenesis induced by ncp BVDV, we examined the activation of all arms of the UPR after infection. We found a significant upregulation on PERK, p-eIF2α, and ATF4 at 12 h postinfection (hpi) (Fig. 3A). Conversely, the expression abundance of ATF6, cleaved ATF6, and p-IRE1 did not display fluctuation during infection, and the spliced form (XBP1s) of XBP1 was not detectable at the mRNA level (Fig. 3B). Consistent with this observation, the qRT-PCR analysis showed that EDEM1, calreticulin, and calnexin mRNA levels were all not affected by ncp BVDV in MDBK cells (Fig. 3C). Interestingly, the mRNA level of the ASNS gene was not significantly upregulated, suggesting that the downstream pathway of the PERK pathway might be not entirely activated. Altogether, these findings reveal that ncp BVDV infection triggers only the PERK branch of the UPR, which occurs earlier than cp BVDV infection. Western blot analysis confirmed this as shown in Fig. S1B.

Two phenotypes of BVDV strongly induced the UPR response at different times, which raised the possibility that UPR machinery contributed to the BVDV pathogenicity. To investigate this, we monitored the process of infectious virion production of two phenotypes of BVDV. Comparing the one-step growth curves of cp and ncp BVDV, we found that their growth trends were generally similar, except that ncp BVDV proliferation was slightly inhibited at 24 h postinfection (hpi) (Fig. 2D and 3D). Taken together, these data indicate that BVDV replication is closely related to the UPR in MDBK cells.

### ER stress benefits BVDV replication.

**FIG 4:**
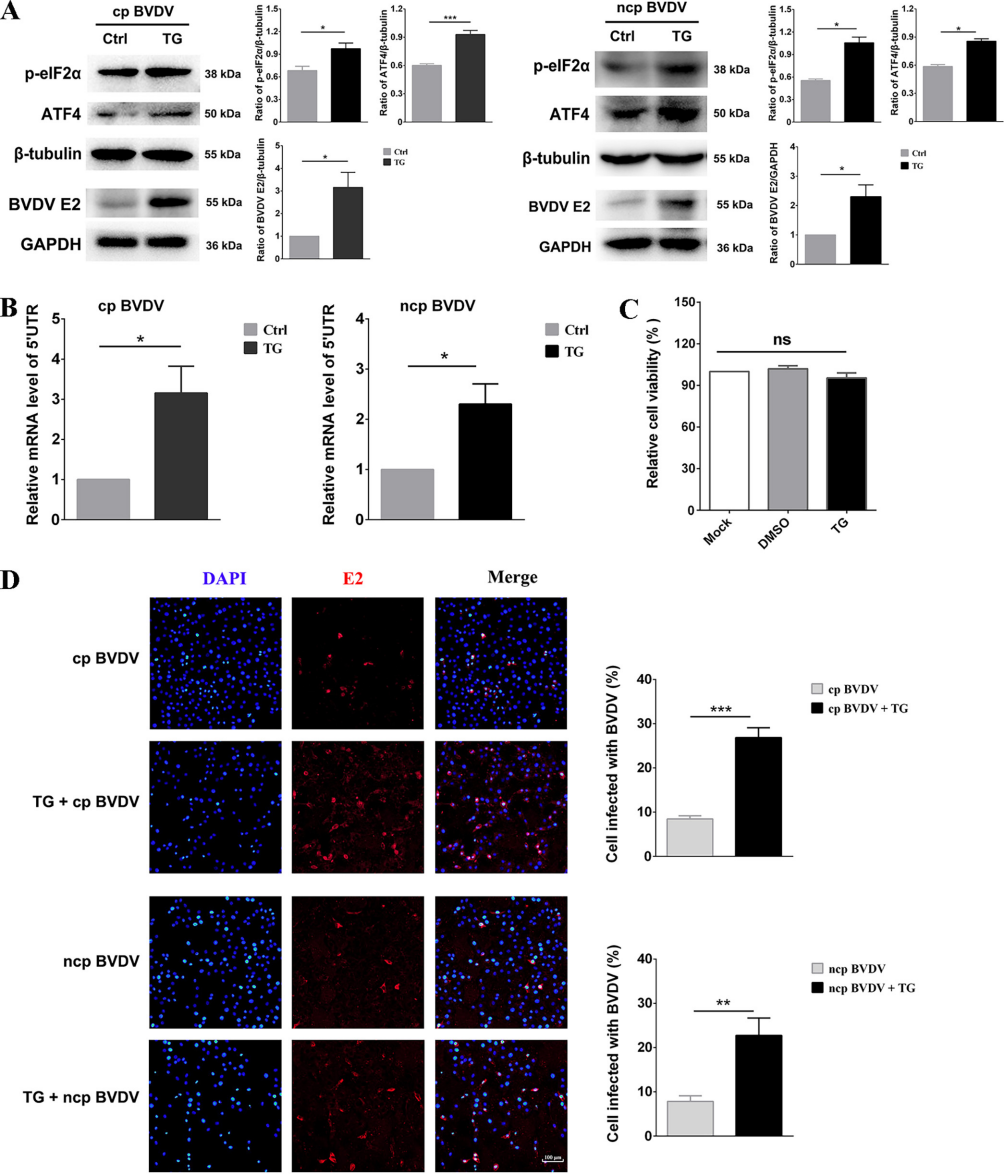
ER stress facilitates BVDV replication in MDBK cells. The MDBK cells were treated with either TG (200 nM) or DMSO for 2 h prior to infection with cp and ncp BVDV and incubated at that concentration until the sample was collected. DMSO is the solvent for TG, and the volume of DMSO used in the control group is the same as that in the TG treatment group. (A) The measurement of p-eIF2α, ATF4, BVDV E2, β-tubulin, or GAPDH expression was conducted by Western blotting. The band intensities in the right columns were presented as fold changes relative to the control group, and the expression of all groups of target proteins was strictly normalized against the loading group. (B) BVDV 5′-UTR expression was assessed by RT-qPCR. The RNA of cp and ncp BVDV-infected cells was detected to determine the expression of 5′ UTR and GAPDH by RT-qPCR. (C) After treatment with TG or DMSO for 26 h, the viability of the cells was assessed with a CCK-8 assay. (D) IFA of cp and ncp BVDV or mock-infected cells treated with TG (200 nM). Viral replication in MDBK cells was assessed by IFA at 24 hpi. Red, BVDV E2 protein; blue, DAPI. At least 100 cells were counted for each group. Scale bars = 100 μm. Student’s *t* test was used for statistical analysis. Values are presented as the mean ± SEM of three experiments that are independent and replicated, and the significance of data is indicated as follows: *, *P < *0.05; **, *P < *0.01; ***, *P < *0.001; ns, no significance.

The virus-induced UPR involved in regulating viral replication was reported previously (33–35). ER stress can suppress the replication of some viruses through the cascade of downstream pathways of the UPR, but many viruses also manipulate the UPR for their own benefit. In order to better understand how ER stress impacted BVDV replication, ER stress inducer TG (200 nM) was added to MDBK cells infected with two phenotypes. The persistent ER stress induced by TG triggered only transient IRE1α and ATF6 activity, but long-lasting PERK activity (36). The results showed that p-eIF2α and ATF4 were further induced in two phenotypes of BVDV-infected MDBK cells by treatment with TG (Fig. 4A). Additionally, compared to the dimethyl sulfoxide (DMSO)-treated group, TG, the ER stress inducer, notably increased the expression level of BVDV E2 and the copy number of BVDV 5′-untranslated region (UTR) RNA (Fig. 4A and B). Given that no obvious change was observed for cell viability after TG treatment, we thought that the promotion of viral reproduction by TG may be independent of cytotoxicity (Fig. 4C). To further determine whether the addition of the ER stress inducer TG facilitated BVDV infection, the viral replication was assessed with an immunofluorescence assay (IFA) based on the signals generated by infectious virions introduced into MDBK cells at 24 hpi. Compared with the control groups, we found an obviously increased number of cp BVDV- and ncp BVDV-infected cells in the TG-treated group (Fig. 4D). Together, the above-described results demonstrate that TG is beneficial for the replication of two BVDV phenotypes in MDBK cells.

### The downstream effector ATF4 of the UPR signaling pathway is critical for BVDV replication.

**FIG 5:**
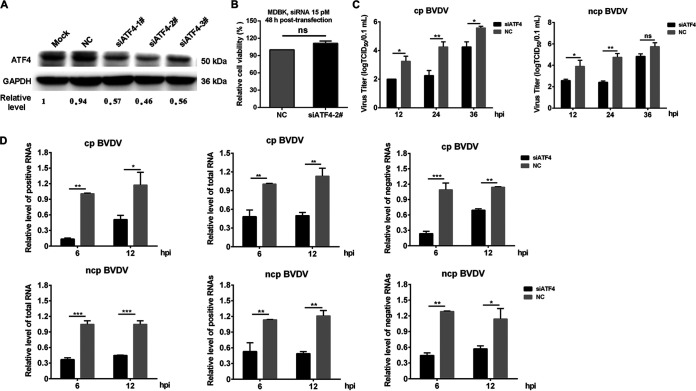
ATF4 is critical for BVDV replication. The scrambled siRNAs (NC) or siRNAs targeting ATF4 (siATF4) were transfected into MDBK cells. (A) When three siRNAs or NC was transfected into cells for 36 h, the knockdown efficiency of ATF4 was assessed by Western blotting. (B) Analysis of the viability of cells transfected with siATF4 and NC using the CCK-8 assay at 36 h. (C) MDBK cells that had been transfected with siRNA for 36 h were infected with cp BVDV or ncp BVDV (MOI, 1). At 12, 24, and 36 hpi, the titer of infectious virus was measured by endpoint dilution assay. (D) At 36 h posttransfection, MDBK cells were infected with cp BVDV or ncp BVDV at an MOI of 1. RT-qPCR was conducted to measure the abundance of viral total RNA and positive- and negative-stranded RNA at 6 and 12 hpi. All RNA species were normalized to GAPDH before comparison with the NC group. Student’s *t* test or two-way ANOVA was used for statistical analysis. Values are presented as the mean ± SEM of three experiments that are independent and replicated, and the significance of data is indicated as follows: *, *P < *0.05; **, *P < *0.01; ***, *P < *0.001; ns, no significance.

ATF4 is an important transcription factor of the CREB/ATF family and regulates the expression of cellular stress-related genes (37, 38). Here, three small interfering RNA (siRNA) molecules targeting ATF4 silencing were designed and transfected into MDBK cells to explore whether ATF4 was involved in BVDV replication. The results showed that ATF4 was precisely targeted and silenced by all siRNAs at 36 h posttransfection, and the effect of sequence-specific knockdown of siATF4-2# was particularly better than that of the other two (Fig. 5A). Meanwhile, the siATF4-2# molecule had no effect on cell viability (Fig. 5B) and thus was used in subsequent infection tests. Viral titers were reduced in cp or ncp BVDV-infected MDBK cells when ATF4 expression was inhibited by siATF4-2# (Fig. 5C), suggesting that UPR effector ATF4 was critical for the efficient proliferation of BVDV.

To further explore whether ATF4 affected the replication of the BVDV genome, we analyzed the effect of silencing ATF4 on the level of viral RNA. In line with the decreased viral load (Fig. 5C), the RNA interference (RNAi) knockdown of ATF4 in MDBK cells significantly inhibited the efficiency of viral RNA synthesis (Fig. 5D). Next, we measured the amount of all RNA species of cp and ncp BVDV in MDBK cells transfected with siATF4 or scrambled negative control (siNC) over a replication cycle. We found that the replication efficiency of BVDV positive- and negative-strand RNA in the ATF4 silencing group was obviously lower than that in the NC group (Fig. 5D). Intriguingly, the replication efficiency of two stands of cp BVDV in the ATF4 silencing group showed a more significant decrease over time than that of ncp BVDV. As described, the transcription factor ATF4 might have a greater effect on cp BVDV replication than ncp BVDV. Collectively, our data reveal that the replication of all BVDV RNA species is inhibited when the expression level of ATF4 is reduced, suggesting that ATF4 may affect the replication of BVDV by regulating some downstream machinery.

### ncp BVDV inhibits ER stress-mediated autophagy by preventing ATF4 from entering the nucleus.

**FIG 6:**
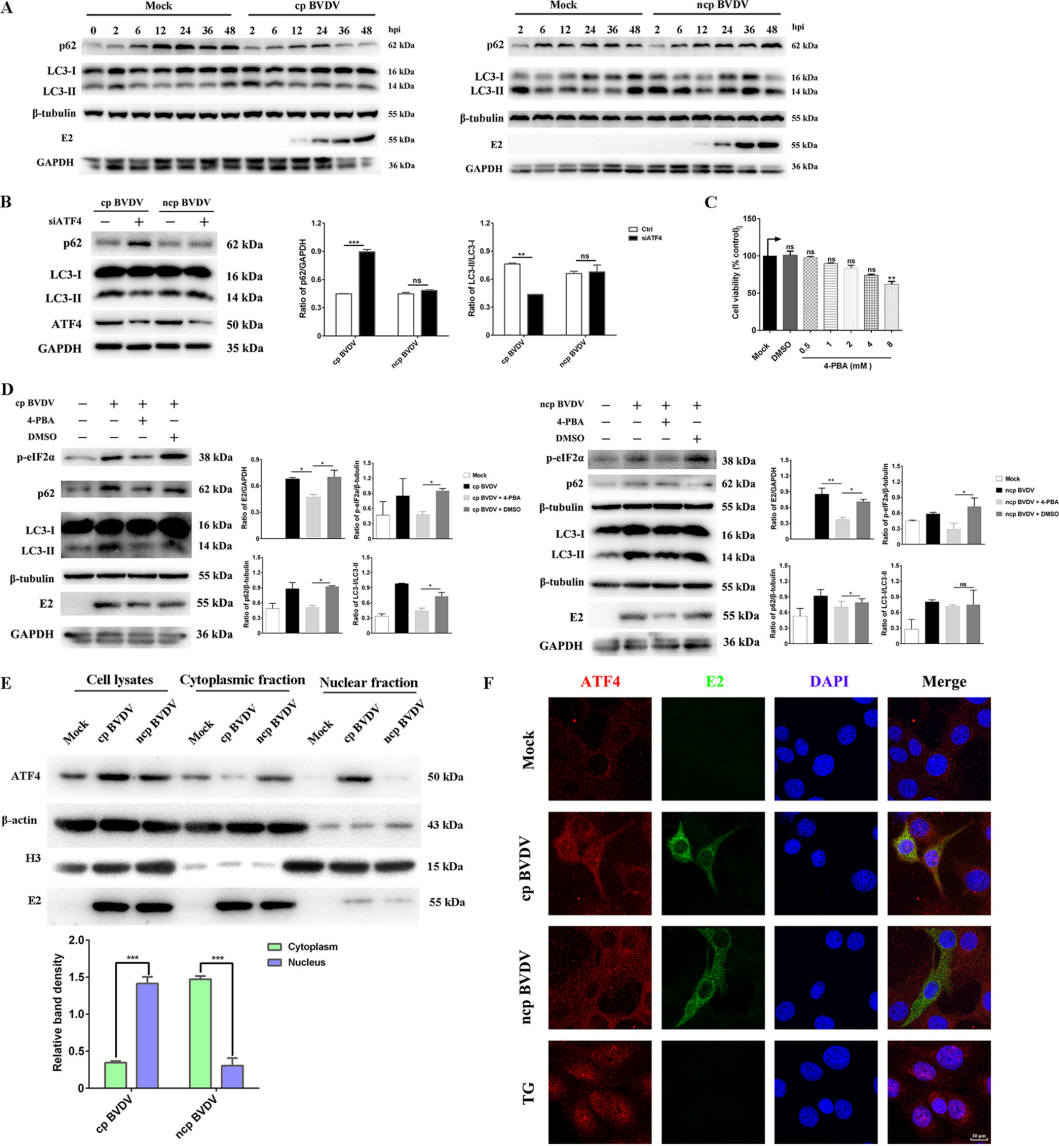
BVDV regulates autophagy by controlling ATF4 nuclear translocation. (A) The conversion of LC3-I to LC3-II and the abundance level of p62 in MDBK cells infected with ncp BVDV or cp BVDV (MOI, 1) at 0, 2, 6, 12, 24, 36, and 48 hpi was assessed by Western blotting. (B) MDBK cells that had been transfected with siRNA-2# for 36 h were infected with cp BVDV or ncp BVDV (MOI, 1). Cells were collected at 24 hpi and then analyzed by Western blotting using the ATF4-, LC3-I-, LC3-II-, p62-, and GAPDH-specific antibodies. (C) The viability of 4-PBA-treated cells was detected with the CCK-8 assay. (D) MDBK cells were pretreated with 4-PBA or DMSO before BVDV infection and harvested at 24 h. Cells were collected and then analyzed by Western blotting using the p-eIF2α-, LC3-I-, LC3-II-, p62-, E2-, GAPDH- and β-tubulin-specific antibodies (E) Cytoplasmic and nuclear fractions of MDBK cells infected with two BVDV phenotypes were extracted and tested for the presence of ATF4. β-Actin and H3 served as the quality control of cytoplasmic and nuclear fractions, respectively (F) Confocal microscopy was used to examine the localization of ATF4 in MDBK cells infected with two BVDV phenotypes for 24 h. TG (200 nM) was added to cells for 2 h as a positive control. Red, ATF4; blue, DAPI; green, E2. Scale bars = 10 μm. Student’s *t* test or one-way ANOVA was used for statistical analysis. Values are presented as the mean ± SEM of three experiments that are independent and replicated, and the significance of data is indicated as follows: *, *P < *0.05; **, *P < *0.01; ns, no significance.

ATF4 controls the transcription of a series of genes that participate in autophagy and apoptosis to maintain ER homeostasis (21). Previous studies have shown that BVDV induces autophagy in host cells and enhances viral replication (39). In our work, we found that UPR promoted the replication of BVDV. Accordingly, we hypothesized that BVDV infection induced UPR downstream effector ATF4 to activate autophagy for its replication. As shown in Fig. 6A, the p62 degradation and conversion of the cytosolic form of LC3 (LC3-I) to the lipidation, membrane-bound form of LC3 (LC3-II) were observed in two phenotypes of BVDV-infected cells but not in control cells at the corresponding time points. These results suggested that the autophagy process activated by the two phenotypes of BVDV was an early event and that the two phenotypes induced autophagy at a similar rate, which was consistent with previous studies (40). To further explore whether ATF4 manipulates BVDV-mediated autophagy, we analyzed the effect of ATF4 silencing on BVDV-activated autophagy. The results showed that the conversion of LC3-I to LC3-II and degradation of p62 induced by cp BVDV infection were inhibited during ATF4 knockdown, but the ncp BVDV-induced autophagy response was not affected (Fig. 6B). Next, we used the chemical chaperone 4-phenylbutyric acid (4-PBA) to define the role of ER stress in the activation of autophagy during BVDV infection. We found that pretreatment with 4-PBA significantly decreased eIF2α phosphorylation in cells in the case of infection with either cp or ncp BVDV, consistent with previous studies showing that 4-PBA relieves ER stress by suppressing the phosphorylation of eIF2α (41, 42). Addition of 4-PBA resulted in a significant decrease of E2 expression, indicating that the eIF2α pathway inhibited the replication of cp and ncp BVDV in MDBK cells (Fig. 6D). Furthermore, the lipidation of LC3 and the protein abundances of p62 were reduced after 4-PBA pretreatment in cp BVDV-infected cells compared with that in cells with added DMSO but remained unchanged in ncp BVDV-infected cells (Fig. 6D). Additionally, the concentration of 4-PBA (2 mM) was noncytotoxic (Fig. 6C), showing that the changes in the expression of autophagy machinery components and E2 were not related to cell viability. Collectively, the above-described results provide strong evidence that cp BVDV infection activates autophagy through ER stress.

Given the critical role of ATF4 for ER stress-induced autophagy in response to BVDV infection, we initially used cytoplasmic-nuclear fractionation to probe for the presence of ATF4. As expected, ATF4 was found mostly in the nuclear fraction of cp BVDV-infected cells. Surprisingly, we found that it appeared to be distributed exclusively in the cytoplasm of ncp BVDV-infected cells (Fig. 6E). Subsequently, immunofluorescence analysis further showed that cp BVDV normally induced ATF4 nuclear translocation. Notably, ATF4 mostly presented in the perinuclear fraction of the ncp BVDV-infected cells (Fig. 6F). In the control group, ATF4 was expressed in low abundance and was present in the peripheral region of the nucleus, but it translocated into the nucleus upon the addition of the ER stress inducer TG. These findings show that ncp BVDV has the ability to prevent ATF4 from entering the nucleus, and it represses the activation of autophagy through the UPR signaling pathway.

### cp BVDV core protein activates the ER stress response via upregulating GRP78 and induces autophagy through the PERK signaling pathway.

**FIG 7:**
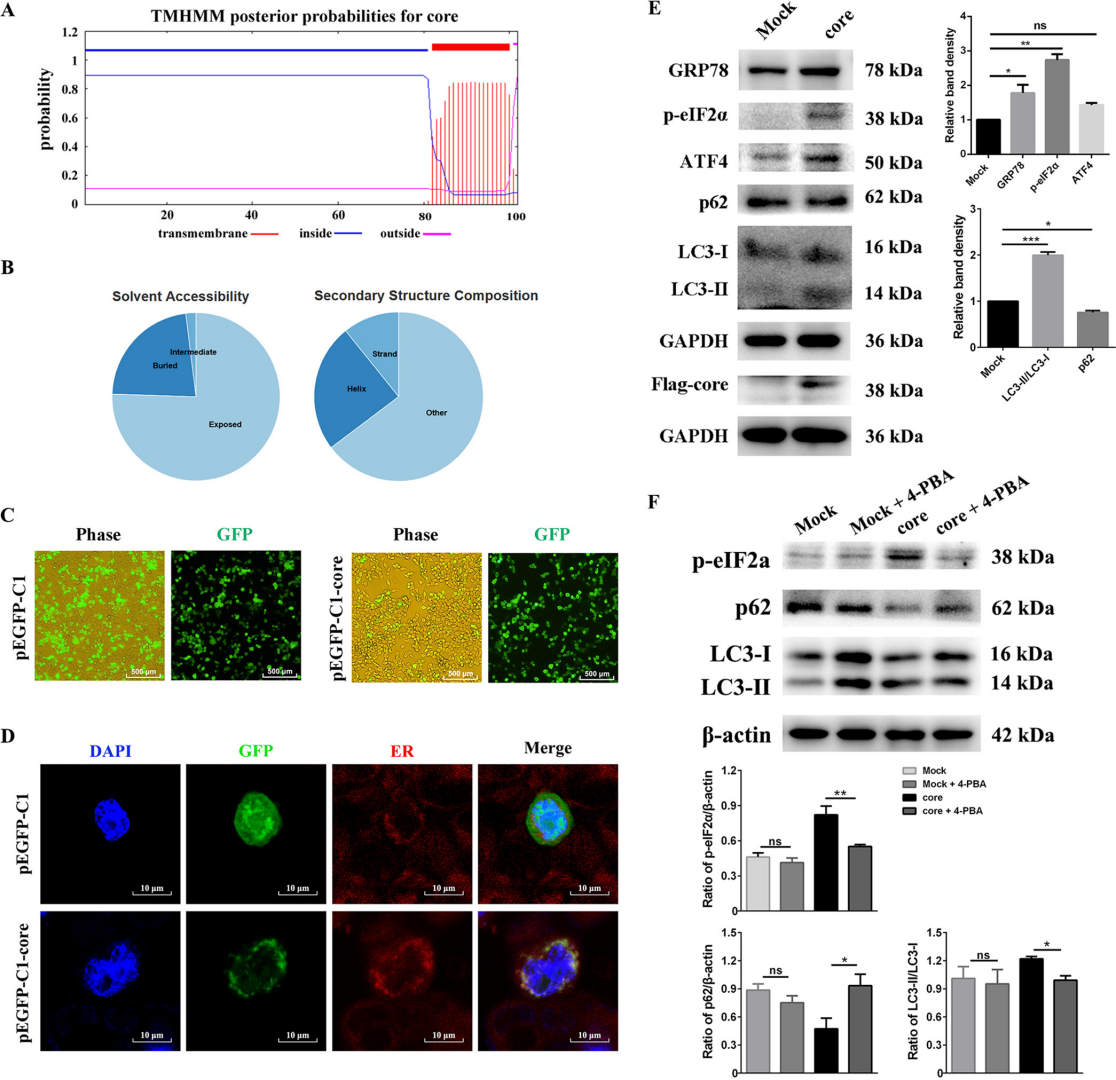
cp BVDV core protein localizes in the ER and induces ER stress-mediated autophagy. (A) Prediction of transmembrane topology of BVDV core protein. (B) Analysis of the solvent accessibility and secondary structure composition (the PredictProtein database) of cp BVDV core protein. (C) 293T cells were transfected with empty vector pEGFP-C1 or recombinant pEGFP-C1-core plasmid. Fluorescence microscopy was used to observe the transfection rate of the core protein in 293T cells at 24 h posttransfection. (D) Confocal microscopy to locate core protein in transfected cells. The transfected cells were stained with ER-Tracker (red) and DAPI (blue), followed by confocal microscopy to detect core protein and ER colocalization. (E) Western blot analysis was performed to measure GRP78, p-eIF2α, ATF4, LC3-I, LC3-II, p62, and core protein expression. GAPDH served as the sample loading control. The band intensities in the right-hand columns were presented as fold changes. (F) 293T cells were treated with 4-PBA (2 mM) after transfection with pEGFP-C1 or pEGFP-C1-core for 22 h and harvested at 24 h posttransfection. Western blotting was conducted to test p-eIF2α, LC3-I, LC3-II, and p62. The band intensities in the bottom columns were presented as fold changes normalized against the loading control. Student’s *t* test or one-way ANOVA was used for statistical analysis. Values are shown as the mean ± SEM of three experiments that are independent and replicated, and the significance of data is indicated as follows: *, *P < *0.05; **, *P < *0.01; ns, no significance.

BVDV and HCV are similar in many respects, including biology, replication cycle, and functional homology of several gene products (43). HCV core protein can activate autophagy through the EIF2AK3 pathway (44). Based on these facts, we speculated that cp BVDV core protein may have the same role as HCV. To verify this speculation, we used the TMHMM software program to predict the transmembrane topology of cp BVDV core protein and then predicted its solubility and intracellular localization using the PredictProtein program (https://predictprotein.org/). The results showed that core protein was a transmembrane protein containing one putative transmembrane domain (TMD) (Fig. 7A and B). Gene ontology for core protein was also predicted by the PredictProtein program, and the results showed core protein targeted the ER membrane with a 32% reliability (GO ID#0006620) indicating that core protein contributes to BVDV-induced ER stress. To study the localization of the core protein, we successfully constructed a Flag-tagged recombinant plasmid, pEGFP-C1-core (Fig. 7C). Compared to the empty vector, a protein band with a molecular mass of approximately 38 kDa was observed in human embryonic kidney (HEK) cells (293T) transfected with pEGFP-C1-core, which was consistent with the predicted protein size (Fig. 7E). Further, we found a large amount of strong speckled green fluorescence in the cytoplasm region of pEGFP-C1-core transfected 293T cells, while the empty vector group was diffusely distributed throughout the cells (Fig. 7C), suggesting that core protein was successfully expressed with high efficiency. To further determine the subcellular localization of the green fluorescent protein (GFP)-core protein, 293T cells transfected with plasmids for 24 h were stained with ER-Tracker. As shown in Fig. 7D, the empty vector GFP fluorescence was distributed widely throughout the cells and did not colocalize with the ER, while GFP-core protein was found to colocalize with the ER-Tracker. Next, to further explore the association between core protein and ER stress, we examined the expression levels of ER stress-related proteins. Interestingly, the abundance of GRP78 increased after being transfected with GFP-core protein, indicating that ER stress was induced by the cp BVDV core protein (Fig. 7E). Furthermore, compared to the GFP control, the expressions of GRP78, ATF4, and p-eIF2α were all significantly upregulated, increased by 1- to 2-, 1- to 2-, and 2- to 3-fold, respectively (Fig. 7E), showing that core protein was involved in the ER stress response via the eIF2α signaling branch. Also, our results demonstrated that cp BVDV core protein induced autophagy by converting LC3-I to LC3-II and degrading p62 (Fig. 7E and F), while the autophagy induced by core protein was significantly suppressed by 4-PBA (Fig. 7F). Taken together, these results indicate that cp BVDV core protein-induced autophagy correlates with the ER stress response.

## DISCUSSION

**FIG 8:**
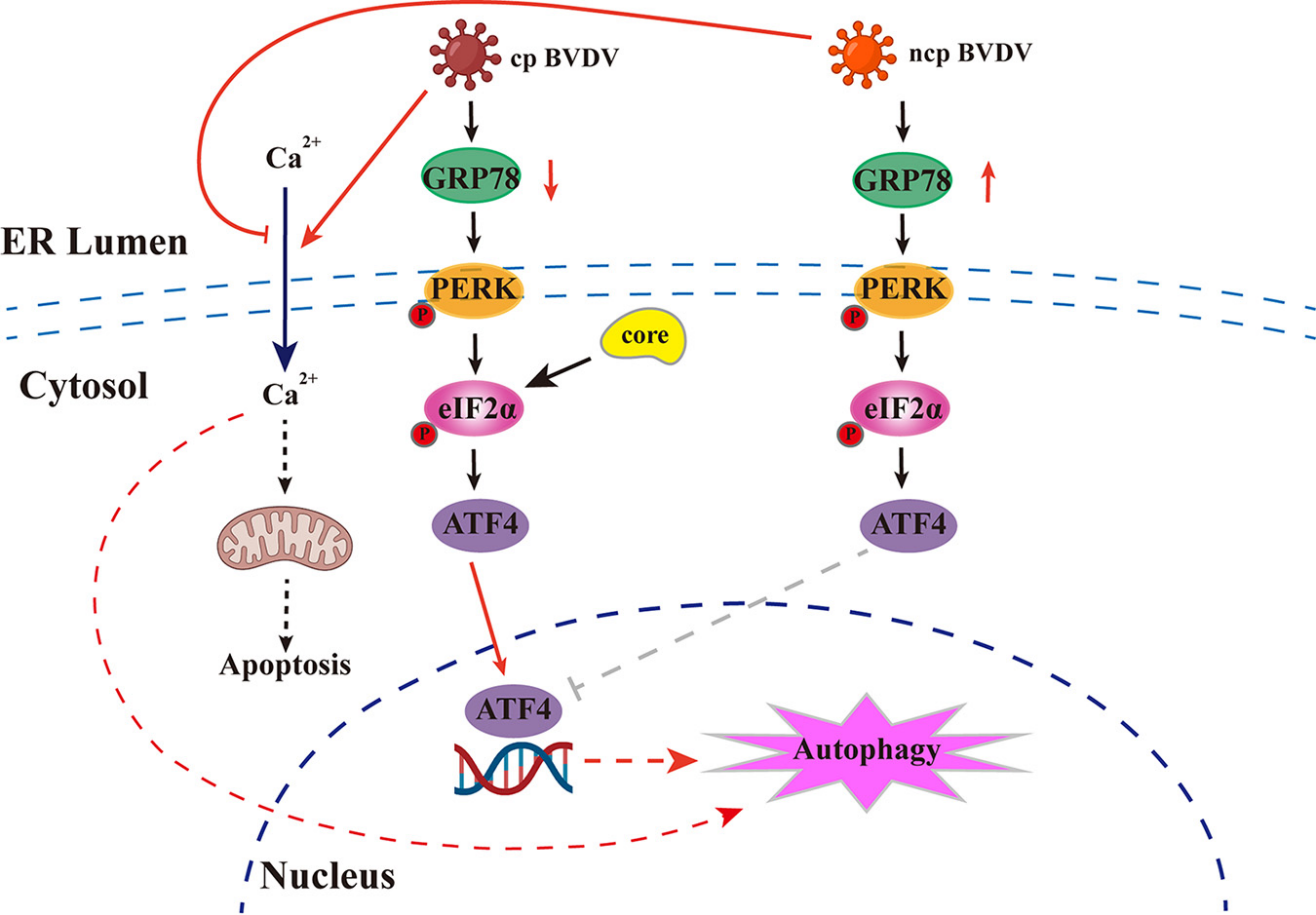
Schematic model of the cellular UPR during BVDV infection. Two phenotypes of BVDV employ diverse regulatory strategies for GRP78 to turn on the PERK-eIF2α-ATF4 branching of UPR at different stages of infection. The activated effector ATF4 enters the nucleus to activate autophagy during cp BVDV infection. However, ncp BVDV infection regulates ATF4 being retained in the cytoplasm and does not activate the autophagy through the UPR. cp BVDV core protein can increase the expression level of GRP78 and induces ER stress-mediated autophagy.

The ER is the membrane platform for replication of *Flaviviridae* members, and the UPR initiated by ER stress implicates the activation of different transcriptional cascades that regulate autophagy, apoptosis, and immunity. In the case of flaviviruses, these underlying mechanisms between UPR and autophagy remain unknown. Here, we report that two phenotypes of BVDV reprogram the cellular UPR to facilitate its own replication utilizing different strategies, which helps to understand how virus-induced ER stress affects flavivirus replication and pathogenesis. Cp BVDV induces or enhances UPR by downregulating GRP78 in an unconventional manner and then regulates ATF4 nuclear translocation to activate autophagy. In contrast, ATF4 in ncp BVDV-infected cells is presented at the perinuclear regions rather than within the nucleus; thus, ncp BVDV does not induce autophagy via eIF2α signaling. Additionally, we first investigated how cp BVDV core protein induces ER stress-mediated autophagy through eIF2α signaling. A schematic model of BVDV manipulation of the UPR to induce autophagy suggested by this study is shown in Fig. 8.

BVDV and other flaviviruses are enveloped RNA viruses that exploit ER membranes to encode large numbers of glycoproteins, which puts great pressure on the folding system of the ER. Here, we unexpectedly found that cp BVDV infection reduced chaperon GRP78 protein abundance by regulating mRNA transcription, and this was not reported in several other ER-tropic viruses. This phenomenon has so far occurred not only in cp BVDV but also in porcine reproductive and respiratory syndrome virus (PRRSV), except that latter targets GRP78 for degradation without affecting RNA levels (45). Also, it was recently reported that SARS-CoV-2 infection slightly inhibited GRP78 levels in cells at 24 hpi, but that expression levels were subsequently upregulated at 48 hpi (46). The downregulated expression of GRP78 may depend on its implicated roles in viral infection, as GRP78 is recognized as a multifunctional regulator of ER homeostasis. In fact, GRP78 is an ER-resident chaperone whose reduced expression is very detrimental to the correct folding of ER-tropic virus proteins. However, it has been reported that the expression of glucose-regulated protein 94 (GRP94) spontaneously increases when GRP78 is knocked down in humans (47). We speculate that cells may upregulate other host chaperones required for nascent viral protein folding. As a master regulator of the cellular UPR, GRP78 maintains the ER stress response in a repressed state, and we found here that ER stress can promote BVDV replication. Therefore, the downregulation of the GRP78 protein level by BVDV infection will further intensify ER stress to benefit viral proliferation. In addition, GRP78 may be an endogenous antiviral factor and exerts antiviral activity by activating the innate immune response (48), suggesting that BVDV may evade host immune responses to maximize proliferation by inhibiting the synthesis of GRP78, and the downregulation of GRP78 levels may be a strategy viruses employ for achieving an optimal cellular environment. In any case, the cytoprotective effect of GRP78 has been documented in a variety of situations, and thus, the low expression of GRP78 may have a positive feedback effect on BVDV proliferation. Interestingly, several studies have found that GRP78 sequesters proapoptotic protein BIK, thereby releasing the inhibition of the antiapoptotic protein BCL-2 and suppressing apoptosis (49). This phenomenon may be related to the induction of apoptosis by cp BVDV infection. However, it remains unknown whether the downregulation of GRP78 is due to specific interactions between viral proteins and host cell factors or is simply a general response to viral replication in the ER.

ER stress generally results from the utilization and disruption of the ER membrane, abnormal accumulation of protein, and virus-induced calcium disturbances. Here, we found that several measures adopted by the UPR to mitigate ER stress might actually help to create a conducive environment for BVDV replication, such as the enlargement of ER volume and the activation of UPR downstream cascades. Specifically, the ER expansion can support the accumulation of glycoprotein and bud virion. Consistent with this, some viruses could establish persistent infections by interacting with UPR (50–52). A host of flaviviruses have evolved strategies to either activate or inhibit particular arms of the UPR to cope with ER stress (53). Previous reports have shown that cp BVDV can activate the PERK branch of the UPR (26), while the other two branches and the interaction of the two phenotypes of BVDV with the ER stress were not studied. In this work, we found that both phenotypes of BVDV only selectively triggered the PERK branch of the UPR without activating the IRE1 or ATF6 pathways. Interestingly, two phenotypes of BVDV activated the PERK pathway at different stages of infection. We speculate that this may depend on the unique characteristics of cp and ncp BVDV, the preferential interaction of the virion with the arms, or the precise interaction of the virus with GRP78 bonded to the sensor. In general, the PERK-eIF2α-ATF4 branch not only inhibits antiviral responses or activates UPR downstream pathways to promote viral replication (54), but also mediates a shutoff of global translation to limit viral replication (34). Compared with cp BVDV, the earlier induction of eIF2α phosphorylation by ncp BVDV could rapidly reduce the peptide load in the ER, so cells would have more time to respond to stimulation. By using this strategy, the pathogenesis of persistent infection or noncytopathic effects caused by ncp BVDV can be illustrated. Additionally, our data showed that cp BVDV strongly induced CHOP expression via ATF4, whereas ncp BVDV showed weak induction of CHOP during infection. CHOP was originally identified as a transcriptional factor that causes apoptosis induced by ER stress (55). Therefore, this might be one of the key factors causing cp BVDV to result in the cytopathic effect *in vitro*. The BVDV-induced increase in CHOP expression levels is consistent with the imbalance of calcium concentration in the cytoplasm. As previously described, calcium is a factor that sensitizes cells to undergo mitochondrion-mediated apoptosis (56). Therefore, the calcium disturbance occurred only in cp BVDV-infected MDBK cells.

In response to stresses, the PERK-eIF2α-ATF4 pathway is essential for global translational attenuation (termed ER-related degradation [ERAD]) upon ER stress and can induce autophagy to engage in the degradation of misfolded protein that cannot be removed by ERAD (57). Previous studies have found that ER stress-mediated autophagy is employed by many viruses for their own replication (58–60), but how it affects BVDV replication has not been well elucidated. In our study, cp BVDV, but not ncp BVDV, activated autophagy via the eIF2α pathway of the UPR, because the suppression of this branch inhibited the formation of complete autophagic flux (Fig. 6). We speculate that ER stress-mediated autophagy may be utilized to balance ER expansion following cp BVDV infection in this situation. Consistent with this observation, only cp BVDV infection can cause calcium disturbance, which can indirectly result in the activation of autophagy. The accumulation of misfolded proteins has been reported to induce inositol 1,4,5-trisphosphate receptor (IP3R_-mediated calcium release, which can not only trigger calcium/calmodulin-dependent protein kinase kinase β (CaMKKβ)) to regulate the mammalian target of rapamycin (mTOR) (61), but also induce the phosphorylation of protein kinase C θ (PKCθ) to activate autophagy (62). Overall, cp BVDV induces ER stress-mediated autophagy, but ncp BVDV does not. It is worth noting that no other group has linked the activation of the UPR with autophagic turnover during BVDV infection. In addition, we further studied the molecular mechanism of ER stress-mediated autophagy induced by cp BVDV, especially the structural protein of BVDV involved in this process. Core protein, a basic and relatively conserved virion nucleocapsid protein among different pestivirus species, can bind to the viral genome (63). Relevant studies have reported that core protein is responsible for a series of cellular signaling pathways and regulates the cellular transcription (64, 65). Nevertheless, the biological properties of cp BVDV core protein remain little known. This work constructed a eukaryotic recombinant expression vector expressing cp BVDV core protein fused with green fluorescent protein (GFP) and Flag tags to analyze its biological function and subcellular localization. We found that the cp BVDV core protein was a transmembrane protein located at the ER, consistent with the core protein of several *Flaviviridae* members (52, 66). Moreover, we provided further evidence that core protein was a major contributor of BVDV to the ER stress response and subsequently mediated autophagy. This is the first study to describe the interaction of BVDV-related proteins with ER stress. However, the core protein is not the key protein responsible for the reduction in GRP78 expression induced by cp BVDV, implying that GRP78 expression may be related to other BVDV proteins. Future research is required to dissect the specific mechanism of the reduced GRP78 expression and how ER stress mediates autophagy. To sum up, the ER not only provides a platform for viral replication but can activate/rearrange ER-related cellular pathways in response to infection. In this way, a comprehensive analysis of the potential linkages between autophagy, apoptosis, and the UPR could help us to better understand the interaction of the *Flaviviridae* family with host cells to develop antiviral drugs.

Transcription factor ATF4 controls multiple signaling pathways that determine cell survival (67), suggesting a complicated position of ATF4 in viral pathogenesis. Few studies have investigated the role of ATF4 in viral replication, and previous studies have mostly concentrated on its role as a DNA transcription factor (34, 54, 59). It was previously shown that the function of ATF4 is related to its cellular localization (68). The ER stress activates nuclear signaling of ATF4 targeting KKLKK (amino acids 280 to 284), allowing its translocation to the nucleus, where ATF4 binds and transcribes relevant downstream target genes (68). Interestingly, we found that ncp BVDV might inhibit ER stress-mediated autophagy by preventing ATF4 entry into the nucleus. Consistent with this observation, when ATF4 was markedly elevated and eIF2α remained phosphorylated, the expression levels of some typical target genes of ATF4, such as CHOP and ASNS, were not affected compared to ncp BVDV infection. Likewise, PRRSV can restrict ATF4 entry into the nucleus by hijacking it to the cytoplasmic viral replication complex (45). All of these findings contribute to a better understanding of virus-host interactions, which may help to elucidate the biology and pathogenesis of BVDV and exploit them as an antiviral strategy. Further studies are required to explore how ATF4 is retained in the cytoplasm by ncp BVDV. Additionally, we also observed that ATF4 played a critical role in the progression of BVDV duplication. Silencing ATF4 expression in MDBK cells caused a significant attenuation in BVDV titer and the synthesis efficiency of all RNA species without any obvious preference, suggesting that ATF4 is an ordinary promoter required for viral RNA synthesis, consistent with a previous report (45), which can be applied to develop a new strategy for bovine viral diarrhea (BVD) treatment. It is worth noting that ATF4 mRNA can be detected in all tissues (69) and represents a vital and promising target for tumors, diabetes, and retinal and neurodegenerative disorders. In addition, a previous study focused on the potential of ATF4 as a clinical therapeutic drug and established a transgenic mouse model (70). Accordingly, ATF4 can be intensively studied as a drug therapy strategy for ATF4-related diseases in humans and animals.

In conclusion, this study investigated the mechanism of the interaction between host cells and viral infection from the perspective of ER stress and revealed how two phenotypes of BVDV interact with the host cells to promote their own replication. Our findings provide new insights into the mechanism of autophagy caused by viral infection via ER stress and reveal an antiviral drug target, ATF4, that contributes to the BVD eradication program.

## MATERIALS AND METHODS

### Cells culture and viruses.

Madin-Darby bovine kidney (MDBK) cells were purchased from the China Veterinary Culture Collection Center (Beijing, China) and cultured in Dulbecco’s modified Eagle medium/nutrient mixture F-12 (F12) (Gibco, Grand Island, NY, USA) supplemented with 10% fetal bovine serum (FBS) (Gibco, Waltham, MA, USA). Human embryonic kidney (HEK) cells (293T) were stored in our laboratory and cultured in RPMI 1640 medium (Gibco, no. 61870044) medium containing 10% FBS at 37°C in a humidified atmosphere of 5% CO_2_.

The BVDV 1-NADL strain (GenBank Accession no. M31182.1) was purchased from the China Veterinary Culture Collection Center (Beijing, China), and the BVDV-BJ175170 strain was isolated from the blood samples of cattle suspected to have BVDV infection and belonged to genotype 1 (GenBank Accession no. MT119454).

### Plasmids construction and transfection.

The pEGFP-C1 vector was stored in our laboratory. The BVDV 1-NADL strain genome was extracted from virus stock with TRIzol reagent. The core gene was amplified from the coding DNA sequence (CDS) region of the genome using gene-specific primers (Table 1) and subcloned into the Bgl II-EcoR I site of the pEGFP-C1 vector. Plasmids were isolated with an Easy Pure HiPure plasmid MaxiPrep kit (EM111, TransGen Biotech, Beijing, China). Next, the recombinant plasmid pEGFP-C1-core was transformed into Escherichia coli BL21(DE3), and the nucleotide sequences of all constructs were identified by DNA sequencing. Lipofectamine 3000 transfection reagent (Thermo Fisher Scientific, Rockford, IL, USA) was used to transfect plasmids into 293T cells according to the reagent instructions. Cells were visualized using blue light with a wavelength of 470 to 480 nm at 24 h posttransfection. When transfected cells successfully expressed green fluorescence, cell lysates were collected and analyzed by Western blotting.

**TABLE 1:** Primers and siRNAs used in this study

Gene primer	Direction^*a*^	Sequence (5′–3′)	Application
BVDV-5′ UTR	F	TAGTCGTCAGTGGTTCGACGC	RT-PCR
	R	CCTCTGCAGCACCCTATCAG	RT-PCR
Core	F	CCCAAGCTTGGGATGTCAGACACGAAAGAAGAGGGAGCAAC	RT-PCR
	R	CGCGGATCCGCGCTAGTGATGATGATGATGATGTCCCATTGTAACTTGAAAC	RT-PCR
GRP78	F	TGCGAAGCCCTATAGCTGAC	RT-qPCR
	R	AGTAGGTGGTACCCAGGTCG	RT-qPCR
EDEM1	F	TCAAGTGTGGCTACGCTACG	RT-qPCR
	R	GAACTCCTTCCAGGGTGACTC	RT-qPCR
ASNS	F	ATCAGGTTGATGATGCAGCGA	RT-qPCR
	R	GCGGCCTTGTAATGGGTCAG	RT-qPCR
Calnexin	F	AAGACCGGAAGCCTGAAGAT	RT-qPCR
	R	CTGGGTCAGGCACATACTCA	RT-qPCR
Calreticulin	F	ATGCCGCTAAGCCTGAAGAC	RT-qPCR
	R	GATCTGCCTGGGTTTCCACT	RT-qPCR
GAPDH	F	ATAGTGGACATCGTCGCCAT	RT-qPCR
	R	CCGTTCTCTGCCTTGACTGT	RT-qPCR
XBP1-474/448	F	AAACAGAGTAGCAGCTCAGACTGC	RT-PCR
	R	TCCTTCTGGGTCCACTTCTGGGAG	RT-PCR
siATF4-1#	F	GCUUUCUCCGGGACAGAUUT	RNAi
	R	AAUCUGUCCCGGAGAAAGCTT	RNAi
siATF4-2#	F	CCAGAAGGUUUACCAACAATT	RNAi
	R	UUGUUGGUAAACCUUCUGGTT	RNAi
siATF4-3#	F	GGAGAUCCAGUAUCUUAAATT	RNAi
	R	UUUAAGAUACUGGAUCUCCTT	RNAi
Scramble	F	UUCUCCGAACGUGUCACGUTT	RNAi
	R	ACGUGACACGUUCGGAGAATT	RNAi
BVDV + RT		GACCTGGATAGGCTGTGTGATACACACGTTATCGGCTTCC	Reverse transcription
BVDV – RT		TTCCGATTAGAGGCGATAGTACTGGCGTCCCTTCCCATCTA	Reverse transcription
SS (+)RNA	F	GCACTGTATGTGAGGGCCGAGA	RT-qPCR
	R	GACCTGGATAGGCTGTGTGATA	RT-qPCR
SS (–)RNA	F	GGTCTTCTCACTTGCATCCATCA	RT-qPCR
	R	TTCCGATTAGAGGCGATA	RT-qPCR

a F, forward; R, reverse. The underlined sequences indicate the enzyme cutting sites.

### Virus titration.

MDBK cells were incubated with BVDV at 37°C for 1 h and cultured with F12 supplemented with 2% FBS after washing off unbound viruses. After repeated freezing and thawing twice of cells infected with virus, the whole-cell culture supernatant was harvested by centrifugation at the indicated time points and then was 10-fold serially diluted to infect MDBK cells in 96-well plates. After being incubated at 37°C for 24 h, cells were fixed with 4% paraformaldehyde (PFA) (Solarbio, China) for 10 min at room temperature, and an immunofluorescence assay (IFA) was performed with mouse anti-BVDV E2 polyclonal antibody and Alexa Fluor 488-labeled goat anti-mouse IgG (H+L) secondary antibody (Beyotime, Shanghai, China) to visualize viral proteins. Viral titers were measured using the Reed-Muench assay and expressed as the logarithm of the infectious dose per 0.1 mL of 50% tissue culture infective dose (TCID_50_/0.1 mL).

### Quantitative reverse transcription-PCR (RT-qPCR).

RNAiso Plus (TaKaRa, Kyoto, Japan) was used to extract the total RNA from MDBK cells, and then the RNA concentration was measured. cDNA was synthesized using a PrimeScript 1st strand cDNA synthesis kit (TaKaRa), and the primer for reverse transcription was random 6-mers according to the reagent instructions. In particular, specific primers (Table 1) were used instead of random 6-mers to detect the BVDV positive- and negative-strand RNA. RT-qPCR was performed in a 20 μL reaction consisting of 0.4 μM forward gene-specific primer, 0.4 μM reverse gene-specific primer (Table 1), 10 μL SYBR green PCR master mix (LS2062, Promega, USA), 1 μL cDNA template, and 8.2 μL RNase-free distilled water (dH_2_O). We performed RT-qPCR amplification at 50°C for 2 min and 95°C for 2 min, followed by 40 cycles of 15 s at 95°C, 15 s at 60°C, and 60 s at 72°C. The mRNA level of the target genes was normalized to the mRNA level of GAPDH, and the results of RT-qPCR were recorded as the fold change using the 2^−ΔΔ^*^CT^* method. Sampling was strictly carried out in triplicate for each reaction.

### XBP1 mRNA splicing assay.

The RNA extraction was performed following the steps described above, and then RNA was reverse transcribed to cDNA. The XBP1 genes were amplified with *Taq* DNA polymerase (Biomed, China). The PCR products were resolved on a 3% agarose gel for electrophoresis. To ensure the more adequate separation of XBP1s and XBP1u, we prepared high-concentration agarose gels and spent sufficient time on nucleic acid electrophoresis. The primer sequences used to amplify XBP1 are listed in Table 1.

### RNA interference assay.

Small interfering RNAs (siRNAs) targeted three different coding regions for ATF4, and control scrambled siRNAs were synthesized by GenePharma (Shanghai, China). The sequences of these siRNAs can be found in Table 1. Lipofectamine RNAiMAX transfection reagent (Thermo Fisher Scientific, Rockford, IL, USA) was used to transfect siRNAs into MDBK cells following the reagent instructions. At 36 h posttransfection, the transfected cells were collected and lysed and then subjected to Western blot analysis to evaluate the silencing effect of siRNAs. For the infection assay, cells were infected with cp or ncp BVDV on the basis of the successful interference of ATF4 expression by siRNA. Subsequently, cell cultures were harvested for the virus titration assay. Additionally, RNA was extracted and reverse transcribed with specific primers (Table 1) for analyzing the abundance of various kinds of RNA by RT-qPCR.

### Cell viability assay.

Cell viability was tested with an enhanced Cell Counting Kit-8 (CCK-8) (Beyotime, Hangzhou, China), which mainly depends on WST-8 reagent. In detail, 5,000 cells per well were seeded in a 96-well cell culture plate in six replicates. Then, 10 μL of the test compound was added to each well and incubated at 37°C in a 5% CO_2_ incubator. At the end of each treatment, the culture medium was replaced with 100 μL medium mixed with 10 μL enhanced CCK-8 solution and then incubated at 37°C for 1 h. Finally, the absorbance of the cell culture plate was measured at 450 nm. The untreated cells were used as controls. All steps in this assay strictly followed the reagent instructions.

### Western blot analysis.

For total protein extraction, radioimmunoprecipitation assay (RIPA) buffer (Solarbio, Beijing, China) supplemented with proteases/phosphatase inhibitor cocktail (Cell Signaling Technology, USA) was used for 30 min at 4°C. The nuclear proteins and cytoplasmic proteins were separated by using the nuclear and cytoplasmic protein extraction kit (Beyotime, Shanghai, China) following the reagent instructions. Cellular proteins were quantified by using a Pierce bicinchoninic acid (BCA) protein assay kit (no. 23227, Thermo Fisher Scientific), and 20 μg of each protein sample was separated by SDS-PAGE, transferred onto polyvinylidene difluoride (PVDF) membranes (Roche), blocked with 5% skim milk at 37°C for 1 to 1.5 h, and then probed with the following primary antibody at 4°C overnight: anti-BVDV E2 (1:1,000, #348) from VMRD (Pullman, WA, USA); anti-PERK (1:1,000, #ab229912) and anti-IRE1 (phospho S724) (1:1,000, #ab205606) from Abcam (Cambridge, UK); anti-ATF4 (1:1,000, #WL02330), anti-ATF6 (1:1000, #WL02407), anti-CHOP (1:1000, #WL00880), and anti-IRE1 (1:1000, #WL02562) from Wanlei Bio (Shenyang, China); anti-p-eIF2α (Ser51) (1:1,000, #3398), anti-BiP/GRP78 (1:1,000, #3177T), anti-LC3A/B (1:1,000, #4108), anti-SQSTM1/p62 (1:1,000, #5114), and anti-ATF6 (D4Z8V) (1:1,000, #65880) from Cell Signaling Technology (Danvers, USA); anti-FLAG (1:5,000, #20543-1-AP), anti-β-tubulin (1:5,000, #10094-1-AP), anti-GAPDH (1:5,000, #60004-1-AP), anti-b-actin (1:5,000, #60008-1-AP), and histone-H3 (1:1,000, #17168-1- AP) from Proteintech Group, Inc. (Rosemont, USA). Next, the membranes were incubated with secondary antibody horseradish peroxidase-conjugated AffiniPure goat anti-mouse IgG (1:5,000, #SA00001-1) or goat anti-rabbit IgG (1:5,000, #SA00001-2) from Proteintech Group, Inc., and then covered and saturated with the enhanced chemiluminescence (ECL) immunoblotting substrate (Thermo Fisher, no. 32209). The protein bands were clearly observed with a Tanon 6200 chemiluminescence imaging workstation (Tanon Science & Technology Co., Ltd., Shanghai, China), and quantified by densitometry using ImageJ software (version 1.50).

### Subcellular localization of the core protein.

After transfection of the vector and recombinant plasmid pEGFP-C1-core into 293T cells using Lipofectamine 3000 (Invitrogen, CA, USA), ER-Tracker red (Beyotime, Shanghai, China) was used to mark the ER for approximately 15 min at 37°C, and 4′,6-diamidino-2-phenylindole (DAPI, 100 ng/mL) (Solarbio, Beijing, China) was used to label the nucleus for 3 min at 25°C. The cells were observed under a laser-scanning confocal microscope (LSCM; Leica SP8, Solms, Germany).

### Immunofluorescence.

MDBK cells were infected with BVDV at a multiplicity of infection (MOI) of 1 when they had grown to 60 to 70% confluence on coverslips in 24-well plates. Then, 4% paraformaldehyde was used to fix the cells (Solarbio, China) at room temperature for 7 to 8 min at the indicated time points, and 1% (vol/vol) Triton X-100 was used for permeabilizing the cells for 10 min. After that, 2% bovine serum albumin (BSA) was used to block the samples, which took 1 to 1.5 h. The plates were subsequently covered with primary antibodies at 4°C for 16 h. Next, the samples were incubated with the appropriate secondary antibodies for 1 h at 25°C. DAPI was used to stain the nuclei for 3 min. Finally, the cells are observed using a Leica SP8 laser scanning confocal microscope (LSCM; Leica, Wetzlar, Germany).

### Transmission electron microscopy (TEM).

After being infected by cp or ncp BVDV, MDBK cells were harvested at 36 hpi and then fixed with 3% glutaraldehyde (pH 7.4). We utilized an H-7500 TEM (Hitachi, Tokyo, Japan) to view and image the sections.

### Flow cytometry.

Flow cytometry was used to measure intracellular Ca^2+^ levels using Fluo-4 AM (Beyotime, Shanghai, China), and MDBK cells were incubated with Fluo4 AM at 37°C for 40 min (evading the light) after digesting and washing 3 times with Hanks balanced salt solution (HBSS). Subsequently, HBSS was added in 5-fold volume with 1% fetal bovine serum, and the cells were incubated at 37°C for an additional 40 min before being washed 3 times with HBSS. In each of the groups, approximately 10,000 or 20,000 cells were detected using a FACSCalibur flow cytometer (BD Biosciences) and analyzed with FlowJo software (version 10.0.7).

### Statistical analysis.

The data in the study were analyzed using Student’s *t* test, one-way analysis of variance (ANOVA), or two-way ANOVA using GraphPad Prism version 6.02 (La Jolla, CA, USA). Error bars represent the means ± standard errors of the means (SEM). A *P* value of less than 0.05 indicates statistical significance.

### Data availability.

All relevant data are presented in the manuscript and its supplemental material.
