# Supplementary material for: Effects of Spatial Expression of Activating Transcription Factor 4 on the Pathogenicity of Two Phenotypes of Bovine Viral Diarrhea Virus by Regulating the Endoplasmic Reticulum-Mediated Autophagy Process
Source: Microbiol Spectr. 2023 Mar 20;11(2):e04225-22. doi: 10.1128/spectrum.04225-22 (PMC10101076; doi:10.1128/spectrum.04225-22)
Supplement: Supplemental file 1 — Fig. S1. Download spectrum.04225-22-s0001.pdf, PDF file, 0.2 MB [file spectrum.04225-22-s0001.pdf]

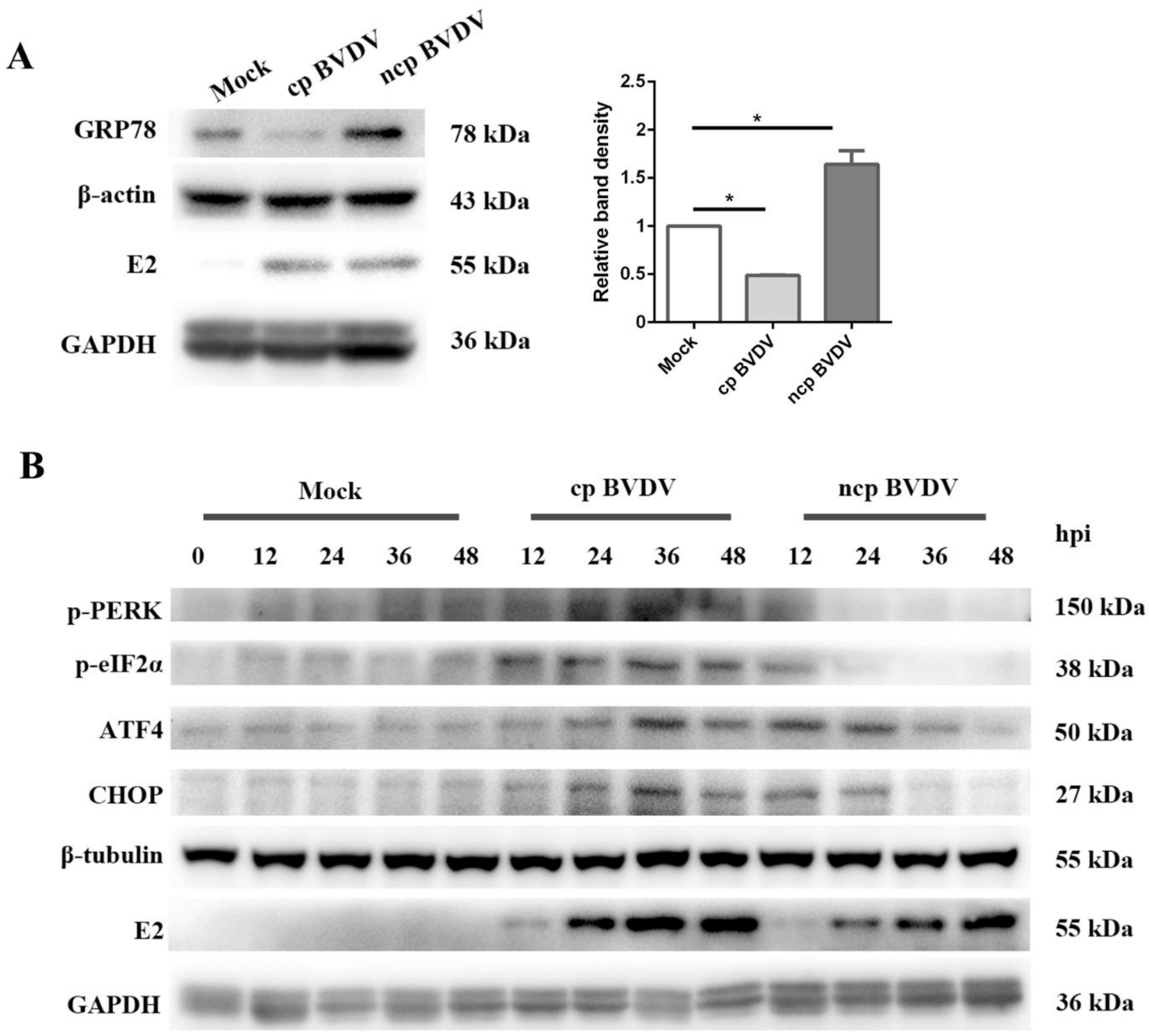

Figure S1 Two phenotypes of BVDV differentially regulate GRP78 expression and induce UPR at different stages of infection. MDBK cells were exposed to cp BVDV (MOI = 1) and ncp BVDV (MOI = 1) and harvested at the indicated time points. (A) Western blot analysis of GRP78 in MDBK cells infected with cp and ncp BVDV at 24 hpi. GAPDH was set for a loading control, and E2 served as an infection indicator. (B) Western blot analysis of proteins related to PERK-eIF2α-ATF4 signaling branch after cp and ncp BVDV challenge. Values are shown as mean ± SEM of three experiments that are independent and replicated, and the significance of data is indicated as follows: *, $P < 0.05$; **, $P < 0.01$; ns, no significance.
